# Primary localized cutaneous nodular amyloidosis presenting as lymphatic malformation: A case report

**DOI:** 10.1515/biol-2021-0076

**Published:** 2021-08-09

**Authors:** Xiujuan Wu, Zongfeng Zhao

**Affiliations:** Department of Dermatology, Shanghai Xuhui Center Hospital, No. 966, Huaihai Road, Shanghai 200031, China; Department of Scientific Research, Shanghai Xuhui Center Hospital, Shanghai 200031, China

**Keywords:** nodular amyloidosis, lymphatic malformation

## Abstract

Primary skin amyloidosis is a chronic skin disease in which amyloid deposits in the normal skin tissues without involving other organs. At present, the causes and mechanisms of morbidity have not been fully elucidated. There are few clinical reports of nodular skin amyloidosis, and the domestic reports are mostly limited cases. This study reported a rare case of a 46-year-old woman with primary localized cutaneous nodular amyloidosis (PLCNA). The patient presented with features of lymphatic malformation, a plexiform nodule of small blisters. Histological examination revealed amyloid deposits involving the superficial and deep dermis with a small number of plasma cells. Further examinations did not reveal evidence of systemic involvement, indicating a PLCNA. The presentation as lymphatic malformation lesions illustrates the importance of clinical pathology. Nodular amyloidosis typically manifests as single or multiple yellow-brown nodules or plaques of a few millimeters to several centimeters. The center of the nodule sometimes shows atrophy and relaxation or forms a bullous. It is recommended to perform a pathological examination to confirm the diagnosis to distinguish it from lymphatic malformation.

## 1 Introduction

Amyloidosis is a general term for diseases caused by amyloid fibrillin deposition. It is divided into several subtypes, including lichen amyloidosis, macular amyloidosis, nodular amyloidosis, and amyloidosis skin pigmentation. Primary localized cutaneous nodular amyloidosis (PLCNA) is a rare disease characterized by a lack of systemic amyloidosis. It could affect the patient’s torso, limbs, face, or genitals. There is no gender difference in the onset of the disease, which is mostly chronic. It sometimes manifests as single or multiple round papules and nodules. Usually, these nodules appear reddish in color, and the skin in the center of some nodules sometimes shows atrophy and relaxation. It is plaque-like atrophy, and some of them have a bull-like appearance [1]. Other atypical subtypes include familial amyloidosis [1], bullous amyloidosis [2], diffuse biphasic amyloidosis [3], poikilothermic amyloidosis [4], and macular amyloidosis with or without hyperpigmentation [5]. PLCNA is the most common among Asians, South Americans, and Middle Easterners [6]. Here we reported a case of PLCNA, providing a basis for elucidation of the etiology, pathogenesis, clinical manifestations, and differential diagnosis of PLCNA.

## 2 Case report

**Figure 1:**
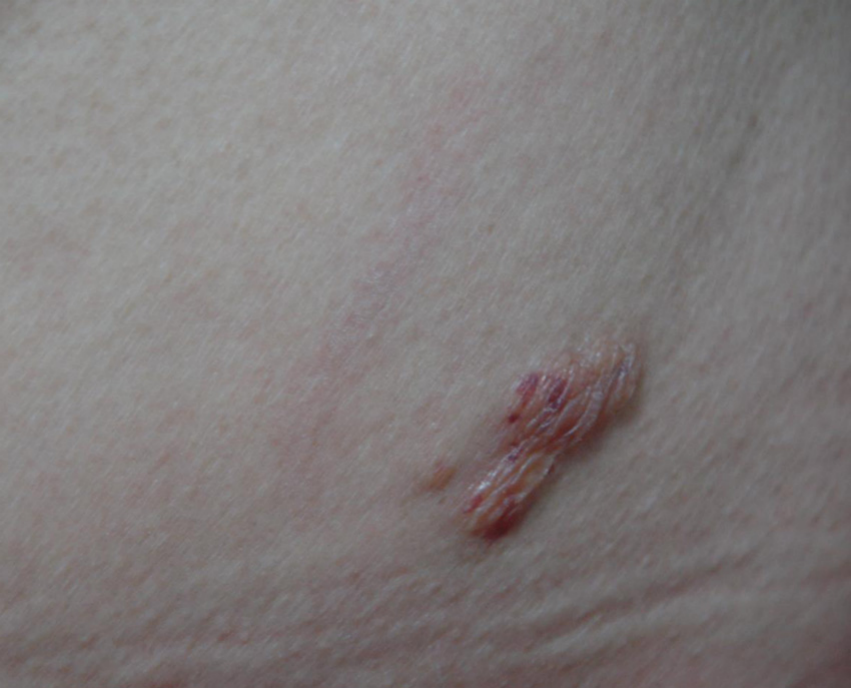
Cluster nodules of small blister-like lymphatic malformation over the left side of the waist.

**Figure 2:**
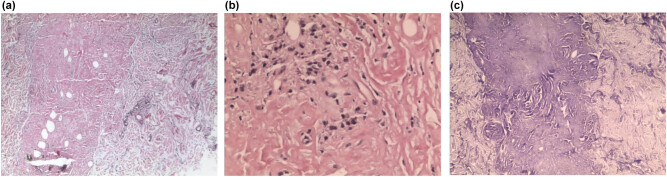
HE staining and methylrosanilinium chloride staining results. (a) A bulk of amorphous, acellular, and faintly eosinophilic material deposit the dermis (H&E, ×100). (b) The infiltration of some plasma cells is shown (H&E, ×200). (c) Methylrosanilinium chloride staining (×100).

**Figure 3:**
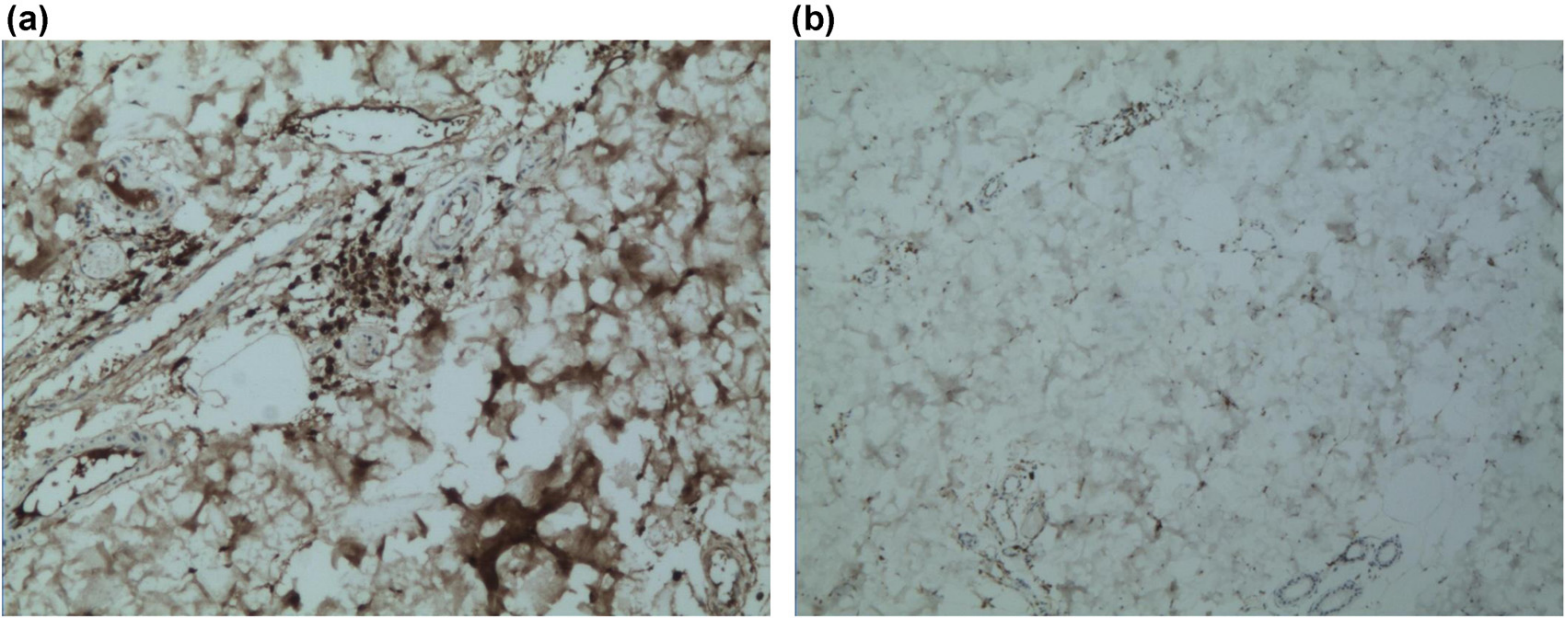
Kappa staining and Lambda staining results. (a) Kappa staining is positive. (b) Lambda staining is positive.

A 46-year-old Chinese woman was admitted to the Department of Dermatology of Shanghai Xuhui Center Hospital in 2014. She had cluster nodules of small blisters on her left waist for 3 years. The blisters were first noticed in 2011 and since then they have expanded and mixed. The patient reported no history of trauma at this site, and no other skin lesions were found by the whole body skin examination. Besides, she did not suffer from pruritus, pain, or other discomforts. Upon examination, we found that the patient had slight erythema on the left side of the waist, which presented as a smooth and loose blister cluster (Figure 1). Because the lesion was blister-like, which is clinically similar to lymphatic malformation, the patient was first diagnosed with lymphatic malformation. A biopsy was performed. A normal appearance of the epidermis was observed in histopathological examination. The dermis appeared as an amorphous aggregation (Figure 2a). A cell-like micro eosinophil was observed, which extended into the deep dermis and was surrounded by plasma cell infiltrates (Figure 2b). The results of methylammonium chloride staining showed purple staining (Figure 2c), consistent with the presentations of amyloid. In addition, both Kappa and Lambda stainings showed positive results (Figure 3). This patient had no family history of amyloidosis. The follow-up physical examination also did not show any clinical or laboratory evidence of systemic amyloidosis or autoimmune diseases, such as weight loss, arrhythmia, giant tongue, dysphagia, or other clinical manifestations. Unfortunately, the medical data on the blood, urine, and bone marrow puncture of the patient were not obtained. There was no clinical evidence of systemic infiltration. The patient was finally diagnosed with PLCNA. Subsequently, the lesion was completely removed by excision. No medication was given thereafter. At approximately one year of follow-up, the patient did not show any evidence of local recurrence or related systemic amyloidosis.

**Informed consent:** Informed consent has been obtained from all individuals included in this study.**Ethical approval:** The research related to human use has been complied with all the relevant national regulations, institutional policies, and in accordance with the tenets of the Helsinki Declaration, and has been approved by the Ethics Committee of Shanghai Xuhui Center Hospital.

## 3 Discussion

Amyloidosis includes a set of diseases, which range from systemic to localized cutaneous subtypes [7]. PLCNA is a rare disease, with only around 60 cases reported by the literature, and the mechanism underlying amyloid deposition is still unknown [8]. It is characterized by extracellular deposition of AL-type amyloid protein in the skin without systemic involvement. It usually presents as macular, lichenoid, nodular, and biphasic [6,9]. However, rare lesions could appear as plaques rather than nodules [6]. There are also rare variants, including cutaneous amyloidosis, cutaneous heterochromia amyloidosis, bullous and sacral forms, and inherited dermatoses associated with amyloidosis. There is a predilection for involvement of the face, particularly the nose and acral sites. The case in our study presented with a rare bullous-like nodular variant. LaChance et al. reported a bullous variant of PLCNA in a 51-year-old man on his third right toe [10]. PLCNA mainly occurs between the ages of 40 and 60, with the same proportion in men and women [11]. It is characterized by focal cutaneous predominant accumulation of light-chain immunoglobulins, secondary to a localized monoclonal proliferation of the plasma cells [12,13,^14^]. In this case, amyloid originated from the immunoglobulin light chain and was associated with dermal plasma cell infiltration, which may penetrate the subcutaneous tissues and blood vessels and might develop into a systemic disease.

Studies have shown a correlation between PLCNA and several autoimmune diseases, the most common ones of which are Sjögren syndrome (about 25% of reported cases) and CREST syndrome [12,13,14,15,^16^]. Yong et al. reported a case of PLCNA in an immunosuppressed patient who underwent a renal transplant for end-stage renal failure caused by immunoglobulin A nephropathy [17]. Another study reported a case of PLCNA from a 33-year-old Japanese man who received cyclosporine, and the patient also had severe atopic dermatitis, bronchial asthma, and cataract since childhood. Another study reported that a 51-year-old man presented with a slightly erythematous, smooth-surfaced papule, with overlying tense blister, on his third right toe, for 2 months. In the study, the lesion was excised completely. With a follow-up for approximately one year, the patient showed no evidence of any local recurrence or related systemic disease. The patient presented with multiple nodules of small blisters like lymphatic malformation lesions.

PLCNA should be differentiated from lymphatic malformation both clinically and pathologically. Lymphatic malformations are benign vascular lesions that arise from embryological disturbances in the development of the lymphatic system. These malformations are thought to result from the abnormal development of the embryonic lymphatics or lymphatic jugular sacs, with failure of these structures to connect or drain into the venous system. Histologically, lymphatic malformations are composed of vascular spaces filled with eosinophilic and protein-rich fluid. A single layer of flattened endothelium lines the walls of lymphatic channels. The fibrovascular vessel walls are of variable thickness, with abnormally formed smooth muscles. Hemorrhage within the cystic spaces is common.

Amyloidosis should be diagnosed based on histological results. Therefore, a tissue biopsy is essential. Stainings, including Congo red, and polarized light help to distinguish amyloid from other pathological fibrils. In this case, HE staining and methylrosanilinium chloride staining of biopsy samples revealed deposition of amyloid and a small number of plasma cell infiltration in the dermis. Kappa and Lambda stainings showed positive results on immunohistochemistry. The patient was finally diagnosed with PLCNA. However, due to insufficient samples, validation by electron microscopy was not performed. Further studies are needed.

## 4 Conclusion

In conclusion, PLCNA is a chronic disease with a generally good prognosis. Therefore, most patients may not receive special treatment if the disease does not affect beauty and health. However, about 4–15% of PLCNA may progress to systemic amyloidosis [18]. Thus, for PLCNA patients, regular follow-up is required.
